# Peripheral Clock System Abnormalities in Patients With Parkinson’s Disease

**DOI:** 10.3389/fnagi.2021.736026

**Published:** 2021-10-01

**Authors:** Tianbai Li, Cheng Cheng, Congcong Jia, Yue Leng, Jin Qian, Hang Yu, Yufei Liu, Nanxing Wang, Yuting Yang, Murad Al-Nusaif, Weidong Le

**Affiliations:** ^1^Liaoning Provincial Key Laboratory for Research on the Pathogenic Mechanisms of Neurological Diseases, The First Affiliated Hospital, Dalian Medical University, Dalian, China; ^2^Department of Psychiatry, Neurology, and Epidemiology and Biostatistics, University of California, San Francisco, San Francisco, CA, United States; ^3^San Francisco VA Medical Center, San Francisco, CA, United States; ^4^Department of Neurology, The First Affiliated Hospital, Dalian Medical University, Dalian, China; ^5^Sichuan Provincial Hospital, Institute of Neurology, Sichuan Academy of Medical Sciences, Chengdu, China

**Keywords:** clock gene, melatonin, Parkinson’s disease, circadian rhythm, sleep-wake disturbances

## Abstract

**Objective:** To evaluate the altered expression of peripheral clock genes, circulating melatonin levels, and their correlations with sleep-wake phenotypes including probable rapid eye movement sleep behavior disorder (pRBD) symptoms in a relatively large population of Parkinson’s disease (PD) patients.

**Methods:** We determined the expression profiles of five principal clock genes, *BMAL1*, *CLOCK*, *CRY1*, *PER1*, and *PER2*, in the peripheral blood mononuclear cells (PBMCs) of PD patients (*n* = 326), and healthy controls (HC, *n* = 314) using quantitative real-time PCR. Melatonin concentration in the plasma of two groups was evaluated by enzyme-linked immunosorbent assay. Then we performed comprehensive association analyses on the PBMCs clock gene expression, plasma melatonin levels and sleep characteristics.

**Results:** Our data showed that the expression levels of *BMAL1*, *CLOCK*, *CRY1*, *PER1*, and *PER2* were significantly decreased in the PBMCs of PD as compared with that of HC (*P* < 0.05). PD patients had reduced plasma melatonin levels compared with HC (*P* < 0.0001). pRBD and excessive daytime sleepiness are common in these PD patients and are associated with the expression levels of all five clock genes (*r* = −0.344∼−0.789, *P* < 0.01) and melatonin concentration (*r* = −0.509∼−0.753, *P* < 0.01). Statistical analyses also revealed that a combination of five clock genes and melatonin could reach a high diagnostic performance (areas under the curves, 97%) for PD comorbid pRBD.

**Conclusion:** This case-control study demonstrates that peripheral *BMAL1*, *CLOCK*, *CRY1*, *PER1, PER2*, and melatonin levels are altered in PD patients and may serve as endogenous markers for sleep and wakefulness disturbances of PD.

## Introduction

Parkinson’s disease (PD) is a common and progressive neurodegenerative disorder that manifests with a broad range of motor and non-motor symptoms (Kalia and Lang, 2015; Schapira et al., 2017). Among a variety of non-motor manifestations of PD, disturbance of sleep and wakefulness is recognized as the most common symptoms affecting as many as 90% of PD patients (Tandberg et al., 1998; Videnovic et al., 2014). Sleep-wake disturbances mainly manifest as poor night-time sleep quality, excessive daytime sleepiness (EDS) and rapid eye movement sleep behavior disorder (RBD) (Li et al., 2021), which ultimately lead to poor quality of life and increased mortality in PD patients (Tandberg et al., 1998; Mattis and Sehgal, 2016).

The sleep-wake cycle is regulated by circadian rhythms (Mattis and Sehgal, 2016). Circadian rhythm is a physiological and behavioral process with a periodicity approximately every 24 h, driven by the endogenous circadian clock in the suprachiasmatic nucleus (SCN), the central circadian pacemaker (Edgar et al., 2012). In addition to sleep-wake disturbances, other circadian-related symptoms including motor activity, visual performance, autonomic dysfunction, and responsiveness to PD medications have also been shown daily fluctuations in PD patients (Videnovic and Willis, 2016; De et al., 2018). Besides, a growing body of research has reported that there exist complicated interactions between the circadian rhythm and dopaminergic systems (Korshunov et al., 2017; Sundaram et al., 2019). All this evidence highlights the complex characteristics of circadian disruption in PD patients, suggesting the need for a reliable and accurate evaluation to identify the main cause of circadian disruption in PD patients.

The circadian rhythm is regulated by a group of clock genes that coordinated their transcription and translation negative feedback loops at a cellular level (Rijo-Ferreira and Takahashi, 2019; Liu et al., 2021). Two of the most important transcription factors are called brain muscle aryl hydrocarbon receptor nuclear translocator-like protein 1 (*BMAL1*) and circadian locomotor output cycles kaput (*CLOCK*), which together activate the transcription of cryptochrome (*CRY*) and period (*PER*) genes (Hergenhan et al., 2020). Clock genes are not only expressed in the central circadian pacemaker but also in most of the peripheral tissues and cells (Hergenhan et al., 2020). It has been shown that the clock machinery is rhythmically expressed in the human peripheral blood mononuclear cells (PBMCs) (Johansson et al., 2016; Zhang S. et al., 2020). Besides, the SCN also transmits its circadian signal to the pineal gland, the organ that secretes melatonin during the dark phase (Wu and Swaab, 2005). Circulating melatonin is a well-accepted marker of endogenous circadian rhythm and its level is altered in patients with PD (Videnovic et al., 2014; Videnovic and Willis, 2016). Therefore, expression levels of clock genes in the PBMCs along with circulating melatonin may provide new avenues to evaluate the circadian abnormalities in PD patients.

There are only a few case studies of circadian rhythm abnormalities in a small number of PD patients in recent years (Fifel, 2017). The objective of this study is therefore to determine whether the expression levels of five principal clock genes, *BMAL1*, *CLOCK*, *CRY1*, *PER1*, and *PER2* in the PBMCs and circulating melatonin levels are altered in PD patients compared to healthy controls (HC). Comprehensive analyses on the associations between the expression levels of PBMCs clock genes, plasma melatonin concentration and the sleep disturbances phenotypes of PD patients have also been performed in this study.

## Materials and Methods

### Participants

A total of 640 participants (326 PD patients and 314 HC) were enrolled between January 2017 and June 2020 (Table 1). Patients with PD in this study were recruited from those who visited the Neurology Department of the First Affiliated Hospital of Dalian Medical University. The PD patients were diagnosed as idiopathic PD according to the Movement Disorder Society Clinical Diagnostic Criteria for PD (Postuma et al., 2015). Among 326 PD patients, 107 were of recent-onset without any PD treatment, the other 219 patients were treated with PD medications. PD disease severity was assessed by Modified Hoehn and Yahr (H-Y) staging (Goetz et al., 2004). Participants of HC were enrolled from the Health Examination Center of Dalian Medical University, showing that they did not have any obvious neurological disorders or non-neurological disorders. Severe depression and psychosis, and other conditions which may affect the circadian rhythms such as melatonin treatment, shiftwork traveling to different time zones within 30 days before the enrollment, were excluded from all the participants. This study has been granted ethical approval by the Ethics Committee of the First Affiliated Hospital of Dalian Medical University (approval number: LCKY2014-29). Written informed consent was obtained from all participants.

**TABLE 1:** Demographics, clinical and sleep characteristics of HC and patients with PD.

**Characteristics**	**Study groups**			**PD patient group (n = 264)**			**PD patient group (n = 264)**		
			***P*-value^c^**			***P*-value^c^**			***P*-value^c^**
	**PD patients (*n* = 326)**	**HC (*n* = 314)**		**Non-pRBD (*n* = 176)**	**pRBD (*n* = 88)**		**Non-EDS (*n* = 104)**	**EDS (*n* = 160)**	
Age^a^	67.43 ± 9.71	66.03 ± 9.24	NS	67 ± 9.82	68.82 ± 9.98	NS	67.17 ± 8.8	67.89 ± 10.58	NS
Gender (M: F)	177: 149	170: 144	NS^d^	95: 81	47: 41	NS^d^	58: 46	85: 75	NS^d^
Disease duration (years)^a^	5.76 ± 4.2	NA	−	5.8 ± 4.3	5.94 ± 4.27	NS	6.09 ± 4.71	5.69 ± 4.06	NS
Hoehn and Yahr score^a^	2.3 ± 1.03	NA	−	2.26 ± 0.74	2.28 ± 0.83	NS	2.25 ± 0.73	2.28 ± 0.8	NS
RBDQ-HK score^b^	22.68 ± 1.33	NA	−	9.5 ± 0.31	48.88 ± 1.9	<0.0001	9.75 ± 0.79	31.14 ± 1.85	<0.0001
ESS score^b^	10.56 ± 0.34	NA	−	8.42 ± 0.38	14.84 ± 0.37	<0.0001	4.79 ± 0.26	14.34 ± 0.24	<0.0001
PSQI score ^b^	10.85 ± 0.31	NA	−	8.72 ± 0.34	15.11 ± 0.29	<0.0001	5.98 ± 0.3	14.06 ± 0.25	<0.0001

*PD, Parkinson’s disease; HC, healthy controls; pRBD, probable rapid eye movement sleep behavior disorder; EDS, excessive daytime sleepiness; M, male; F, female; RBDQ-HK, RBD questionnaire–Hong Kong; ESS, Epworth Sleepiness Scale; PSQI, Pittsburgh Sleep Quality Index; NS, not significant; NA, not analysed.*

*^*a*^Data are expressed as mean ± SD.*

*^*b*^Data are expressed as mean ± SEM.*

*^*c*^Unless otherwise indicated, generated by the Kruskal-Wallis test.*

*^*d*^Indicates generated by the chi-square test.*

### Sleep-Related Measurement Scales

The severity of sleep and wakefulness disturbances in PD patients was assessed by the sleep-related measurement scales including the RBD questionnaire–Hong Kong (RBDQ-HK), Epworth Sleepiness Scale (ESS), and Pittsburgh Sleep Quality Index (PSQI). The RBDQ-HK is a Chinese instrument for screening and modifying the severity of RBD (Dauvilliers et al., 2008). Since patients in this study had not received a polysomnography diagnosis, those who have the RBDQ-HK score of more than 18 (the cutoff point) were determined as the probable RBD (pRBD) (Shen et al., 2014; Wong et al., 2016). Daytime sleepiness was measured by the Chinese version of the ESS. A total score ≥ 10 indicates EDS (Videnovic et al., 2014). PSQI was used in the assessment of the night sleep quality of participants (Mollayeva et al., 2016).

### Blood Sampling, Plasma, and Peripheral Blood Mononuclear Cells Separation

When the blood sampling, all the participants (PD patients and HC) were not strictly shielded from external light with the constant ambient temperature at 21 ± 2°C. Fasting peripheral blood samples (2 mL) were collected by direct venipuncture at 6–7 a.m. The plasma was aliquoted (800 μL) into a sterile tube and stored at −80°C. PBMCs were separated from the Peripheral Lymphocyte Separation Medium (HAOYANG, Tianjin, China) by centrifugation at 450 g for 20 min and were stored at −80°C.

### Peripheral Blood Mononuclear Cells mRNA Extraction and Quantification

Total RNAs from the PBMCs were extracted utilizing the mirVana mRNA Isolation Kit (Ambion, Carlsbad, CA, United States). The target five clock genes mRNA levels were then measured by quantitative real-time PCR (qRT-PCR). The PCR reactions were executed using ABI 7500 fast real-time PCR system (Applied Biosystems, Foster City, CA, United States) in a total volume of 20 μL for each reaction. *GAPDH* gene was used as the housekeeping gene. The specific primers targeting PBMCs *BMAL1*, *CLOCK*, *CRY1*, *PER1*, and *PER2* are presented in Supplementary Table 1. After 94°C for 30 s, the experimental reaction consisted of 40 cycles of 94°C for 5 s and 60°C for 34 s, and the target genes were detected by the fluorescent dye SYBR Green I (TransGen, Beijing, China). The target genes expression was determined using the 2^–delta Ct^ method.

### Plasma Melatonin Measurement

A total of 309 plasma samples from the participants in this study (156 from HC and 153 from PD) were separated simultaneously with the extraction of PBMCs. Plasma melatonin concentration was evaluated using the Human Melatonin enzyme-linked immunosorbent assay (ELISA) Kit (Elabscience, Wuhan, China) according to the manufacturer’s instructions. The concentration of melatonin in the plasma was finally determined by comparing the OD values of the samples to the standard curves. The analytical sensitivity of the assay was 9.38 pg/mL. The intra-assay coefficients of variation for the kits were 9.4% (PD) and 9.5% (HC), and the inter-assay variation coefficients were 9.2% (PD) and 9.5% (HC).

### Statistical Analysis

Data were examined for normality using the Shapiro-Wilk test. Quantitative data were expressed as mean ± standard error (SE) for each group. We analyzed group differences using the Kruskal-Wallis and non-parametric Mann-Whitney *U*-test. Spearman’s coefficients were calculated to evaluate bivariate associations between the demographic, disease severity, sleep characteristics, plasma melatonin concentration and the expression levels of clock genes. Partial correlation analyses were utilized to adjust for age, sex, and pharmacotherapy. Receiver operating characteristic (ROC) curves and areas under the curves (AUC) were used to evaluate the diagnostic performance of the potential biomarkers. The alterations of the clock genes levels and melatonin concentration were evaluated using two-way ANOVA for the effect of sunlight. Statistical analyses were performed with the SPSS software version 22.0 (SPSS Inc., Chicago, IL, United States). A *P*-value < 0.05 was considered as statistical significance.

## Results

### Characteristics of the Study Population

In this study, we collected a total of 640 PBMCs samples: 326 from patients with idiopathic PD and 314 from HC. All participants enrolled in this study were Chinese. Demographics, disease status, and sleep characteristics of the study cohort are outlined in Table 1. No significant difference in sex or age was found between the PD and HC groups. In the 264 PD patients (80.98%) who returned the sleep-related questionnaires, RBDQ-HK scores ranged from 2 to 91, with 88 PD patients (33.33%) classified as patients with pRBD (RBDQ-HK score greater than 18). 160 patients (60.6%) had EDS, as defined by an ESS score of more than 9. PD patients with pRBD presented more serious EDS and poorer sleep quality (*P* < 0.0001). Similarly, patients with EDS also had more severe RBD symptoms and poorer sleep quality (*P* < 0.0001). Demographics, disease duration, and H-Y scores were not significantly different in the subgroups of PD patients (Table 1).

### Altered Expressions of *BMAL1*, *CLOCK*, *CRY1*, *PER1*, and *PER2* Genes in the Peripheral Blood Mononuclear Cells of Parkinson’s Disease Compared With Healthy Controls

**FIGURE 1:**
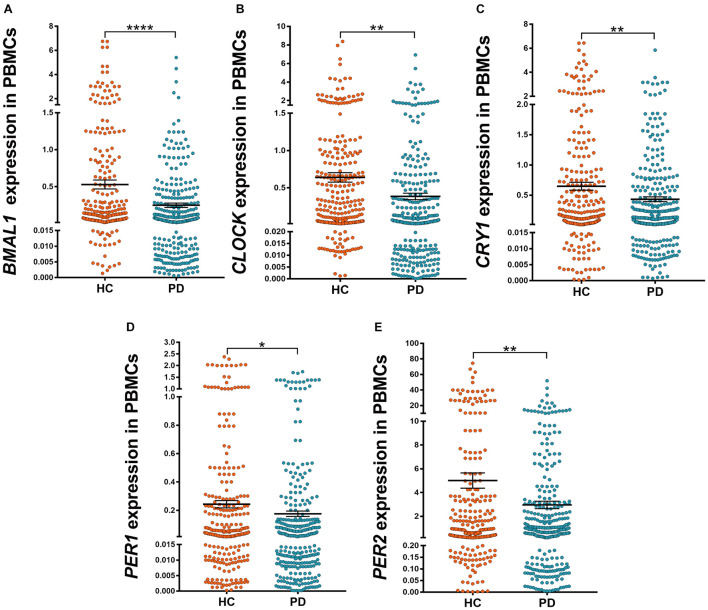
Scatter plots of *BMAL1*, *CLOCK*, *CRY1*, *PER1*, and *PER2* relative mRNA expression levels in the PBMCs of HC (*n* = 314) and PD (*n* = 326). **(A–E)** Significantly lower levels of *BMAL1* (**A**, *P* < 0.0001), *CLOCK* (**B**, *P* < 0.01), *CRY1* (**C**, *P* < 0.01), *PER1* (**D**, *P* < 0.05) and *PRY2* (**E**, *P* < 0.01) were seen in PD patients compared to those in HC. Horizontal bars represent mean and SE values. *P < 0.05, **P < 0.01, and ****P < 0.0001.

We determined five clock genes (*BMAL1*, *CLOCK*, *CRY1*, *PER1*, and *PER2*) mRNA levels in the PBMCs of PD patients (*n* = 326) and HC (*n* = 314) by qRT-PCR. Our data showed significantly lower levels of *BMAL1* (*P* < 0.0001), *CLOCK* (*P* < 0.01), *CRY1* (*P* < 0.01), *PER1* (*P* < 0.05), and *PRY2* (*P* < 0.01) in PD than those in HC (Figures 1A–E).

**FIGURE 2:**
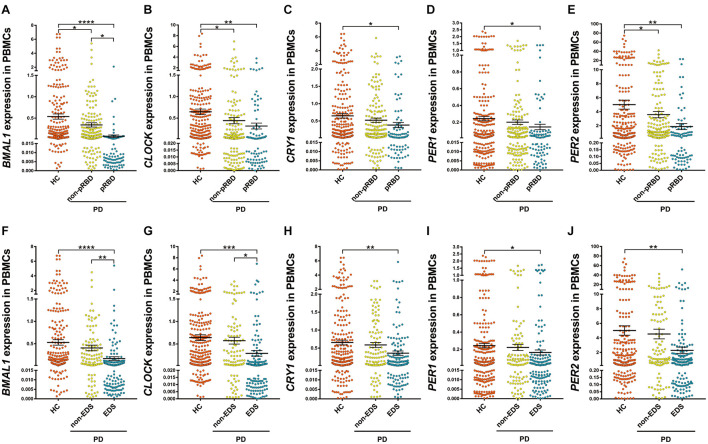
Scatter plots of *BMAL1*, *CLOCK*, *CRY1*, *PER1*, and *PER2* relative mRNA expression levels in the PBMCs of HC and different subgroups of PD. **(A–E)** PD patients with pRBD (*n* = 88) had significantly lower expression levels of five clock genes compared with those in HC (*n* = 314, *BMAL1*, *P* < 0.0001; *CLOCK*, *P* < 0.01; *CRY1*, *P* < 0.05; *PER1*, *P* < 0.05; *PER2*, *P* < 0.01, respectively), and had decreased level of *BMAL1* compared with that of PD patients without pRBD (*n* = 176, *P* < 0.05). **(F–J)** Expression levels of all five clock genes in PD patients with EDS (*n* = 160) were significantly lower than that of HC (*n* = 314, *BMAL1*, *P* < 0.0001; *CLOCK*, *P* < 0.001; *CRY1*, *P* < 0.01; *PER1*, *P* < 0.05; *PER2*, *P* < 0.01, respectively). Significantly differences of *BMAL1* (*P* < 0.01) and *CLOCK* (*P* < 0.05) expression levels were seen between the subgroups of PD patients with EDS and without EDS. Horizontal bars represent mean and SE values. *P < 0.05, **P < 0.01, ***P < 0.001, and ****P < 0.0001.

Among PD patients, those with pRBD (*n* = 88) had significantly lower expression levels of all five clock genes compared with those in HC (*n* = 314, *P* < 0.05, Figures 2A–E), in particular, they had lower expression levels of *BMAL1* when comparing to the PD patients without pRBD (*n* = 176, *P* < 0.05, Figure 2A). PBMCs *BMAL1* (*P* < 0.05), *CLOCK* (*P* < 0.05), and *PER2* (*P* < 0.05) levels in PD patients without pRBD were also decreased markedly compared with those in HC (Figures 2A,B,E). Similarly, expression levels of all five clock genes in PD patients with EDS (*n* = 160) were significantly lower than those in HC (*n* = 314, *P* < 0.05, Figures 2F–J). Significant differences in *BMAL1* (*P* < 0.01) and *CLOCK* (*P* < 0.05) expression levels were seen between the subgroups of PD patients with EDS and without EDS (Figures 2F,G). However, when comparing the five clock genes expression levels in the subgroups of patients without EDS and HC, no significant difference was observed.

We then analyzed associations between the expression levels of clock genes and the clinical characteristics of PD patients. Correlation coefficients showed significant associations among the expression levels of five clock genes (Table 2). Expression levels of *CLOCK* (*r* = −0.143, *P* < 0.05) and *CRY1* (*r* = −0.12, *P* < 0.05) were inversely associated with the age of the PD patients (Table 2). However, disease duration (years after the onset of disease symptoms) and disease severity (the H-Y score) in the PD group were not significantly correlated with the expression levels of the five clock genes (correcting for age, sex, and pharmacotherapy, Table 2). In the analysis of the relationships between PD pharmacotherapy and clock genes expressions, no significant difference of the five clock genes levels was found among the four different pharmacotherapy subgroups of PD (correcting for age and sex, Supplementary Figure 1A).

**TABLE 2:** The correlation coefficients between the expression levels of clock genes in the PBMCs, plasma melatonin concentration, the age and sex of the PD patients, and HC.

		***CLOCK***	***CRY1***	***PER1***	***PER2***	**Melatonin**	**Age**	**Sex**
*BMAL1*	HC	0.255**	0.279**	0.241**	0.215**	0.223**	–0.002	0.036
	PD	0.659**	0.654**	0.537**	0.559**	0.588**	–0.058	0.008
*CLOCK*	HC		0.35**	0.443**	0.321**	0.381**	0.019	–0.036
	PD		0.557**	0.556**	0.657**	0.477**	−0.143*	–0.023
*CRY1*	HC			0.531**	0.641**	0.522**	–0.05	–0.035
	PD			0.533**	0.479**	0.448**	−0.12*	–0.023
*PER1*	HC				0.332**	0.365**	0.001	–0.022
	PD				0.54**	0.308**	–0.075	–0.041
*PER2*	HC					0.415**	–0.021	0.013
	PD					0.381**	–0.082	–0.042
Melatonin	HC						–0.0442	–0.023
	PD						–0.057	–0.019
Age	HC							–0.022
	PD							0.029

*P < 0.05. **P < 0.01.

### Altered Concentration of Melatonin in the Plasma of Parkinson’s Disease Compared With Healthy Controls

**FIGURE 3:**
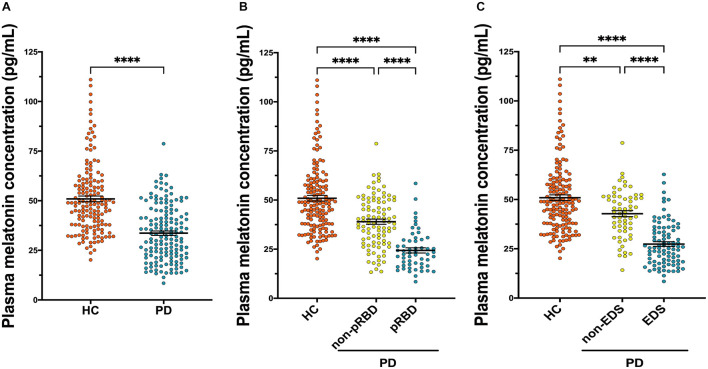
Scatter plots of melatonin concentration in the plasma of HC (*n* = 156) and PD (*n* = 153). **(A)** Plasma melatonin concentration was significantly decreased in patients with PD than in HC (*P* < 0.0001). **(B)** PD patients with pRBD (*n* = 56) had significantly lower melatonin concentration compared with those in HC (*n* = 156, *P* < 0.0001) and PD patients those without pRBD (*n* = 97, *P* < 0.0001). A significant difference in melatonin concentration was found between HC and PD without pRBD (*P* < 0.0001). **(C)** Alterations of plasma melatonin concentration were found between PD with EDS (*n* = 92) and PD without EDS (*n* = 64, *P* < 0.0001), and both of two groups have significant lower melatonin concentration when comparing to HC (PD with EDS, *P* < 0.0001; PD without EDS, *P* < 0.01). ^∗∗^*P* < 0.01 and ^****^P < 0.0001.

Results from ELISA show that plasma melatonin concentration was significantly decreased in patients with PD than in HC (*P* < 0.0001, Figure 3A). PD patients with pRBD (*n* = 56) had significantly lower melatonin concentration compared with those in HC (*n* = 314, *P* < 0.0001) and PD patients without pRBD (*n* = 97, *P* < 0.0001). Besides, there is a significant difference between melatonin concentration in HC and PD patients without pRBD (*P* < 0.0001, Figure 3B). Similar alterations of plasma melatonin concentration were also found between EDS group (*n* = 92) and negative EDS group (*n* = 64) in PD patients (*P* < 0.0001), and both of two groups have significant lower melatonin concentration when comparing to HC (PD with EDS, *P* < 0.0001; PD without EDS, *P* < 0.01; Figure 3C). Spearman and partial correlation analyses show that there are no significant associations between melatonin concentration, age, sex, disease duration or disease severity (Tables 2, 3). Furthermore, plasma melatonin concentration in four different pharmacotherapy subgroups of PD didn’t reach a statistical difference (Supplementary Figure 1B).

**TABLE 3:** The correlation coefficients between the expression levels of clock genes in the PBMCs, sleep, and disease characteristics of the PD patients.

	***CLOCK***	***CRY1***	***PER1***	***PER2***	**Melatonin**	**RBDQ-HK**	**ESS**	**PSQI**	**Disease duration^a^**	**H-Y score^a^**
*BMAL1*	0.658**	0.677**	0.531**	0.565**	0.588**	−0.789**	−0.644**	−0.739**	0.007	0.019
*CLOCK*		0.578**	0.538**	0.659**	0.477**	−0.527**	−0.513**	−0.583**	0.031	–0.023
*CRY1*			0.527**	0.481**	0.448**	−0.568**	−0.528**	−0.532**	0.014	0.003
*PER1*				0.531**	0.308**	−0.396**	−0.344**	−0.431**	0.006	–0.012
*PER2*					0.381**	−0.447**	−0.366**	−0.447**	0.087	0.009
Melatonin						−0.579**	−0.509**	−0.753**	0.024	–0.022
RBDQ-HK^a^							−0.59**	−0.628**	–0.003	–0.023
ESS^a^								0.857**	0.03	0.003
PSQI^a^									0.049	0.057
Disease duration										0.495**

*RBDQ-HK, RBD questionnaire–Hong Kong; ESS, Epworth Sleepiness Scale; PSQI, Pittsburgh Sleep Quality Index.*

^*a*^Partial correlation analysis, correcting for age, sex, and pharmacotherapy. **P < 0.0.

### Associations Between the Expression Levels of Clock Genes, Plasma Melatonin, and Sleep-Wake Disturbances in Parkinson’s Disease Patients

We then determined the correlations between the severity of pRBD (RBDQ-HK score), the severity of daytime sleepiness (ESS score), self-reported sleep quality (PSQI score), PBMCs levels of five clock genes, and plasma melatonin levels of PD patients (correcting for the age, sex, and pharmacotherapy). Our results showed that the RBDQ-HK, ESS, and PSQI scores completed by the PD patients (*n* = 264) were inversely associated with the expression levels of all five clock genes (*r* = −0.344∼−0.789, *P* < 0.01) and plasma melatonin (*r* = −0.509∼−0.753, *P* < 0.01, Table 3).

**FIGURE 4:**
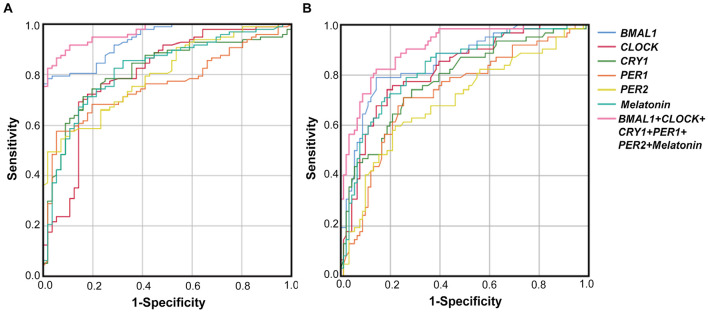
Receiver operating characteristic (ROC) curves of *BMAL1*, *CLOCK*, *CRY1*, *PER1*, *PER2* and melatonin levels for PD with pRBD vs. PD without pRBD, and those for PD with EDS vs. PD without EDS. **(A)** The AUCs values of *BMAL1*, *CLOCK*, *CRY1*, *PER1*, *PER2*, melatonin, and the combination of five clock genes and melatonin for pRBD were 0.94 (*BMAL1*, 95% CI, 0.9–0.97, *P* < 0.05), 0.8 (*CLOCK*, 95% CI, 0.73–0.88, *P* < 0.05), 0.81 (*CRY1*, 95% CI, 0.74–0.88, *P* < 0.05), 0.76 (*PER1*, 95% CI, 0.68–0.84, *P* < 0.05), 0.8 (*PER2*, 95% CI, 0.73–0.87, *P* < 0.05), 0.82 (melatonin 95% CI, 0.75–0.89, *P* < 0.05), and 0.97 (combined five clock genes and melatonin, 95% CI, 0.94–0.99, *P* < 0.05), respectively. **(B)** The AUCs values for EDS were 0.86 (*BMAL1*, 95% CI, 0.8–0.92, *P* < 0.05), 0.82 (*CLOCK*, 95% CI, 0.75–0.89, *P* < 0.05), 0.78 (*CRY1*, 95% CI, 0.71–0.86, *P* < 0.05), 0.73 (*PER1*, 95% CI, 0.65–0.81, *P* < 0.05), 0.7 (*PER2*, 95% CI, 0.61–0.78, *P* < 0.05), 0.83 (melatonin, 95% CI, 0.76–0.9, *P* < 0.05), and 0.91 (combined five clock genes and melatonin, 95% CI, 0.86–0.96, *P* < 0.05), respectively.

The performance of PBMCs clock genes and plasma melatonin for the diagnosis of PD comorbid pRBD and EDS were evaluated by the AUC values based on the ROC curve analysis. The results showed that AUCs of the combination of five clock genes and melatonin for pRBD were 0.97 (95% CI, 0.94–0.99, *P* < 0.01), which outperformed those of five clock genes and melatonin alone (Figure 4A). Similarly, the AUC value of the combined five clock genes with melatonin was 0.91 (95% CI, 0.86–0.96, *P* < 0.01) for EDS, which reached a higher performance compared to these factors alone (Figure 4B).

### Altered Clock Genes and Melatonin With the Changes of Sunlight Intensity

**FIGURE 5:**
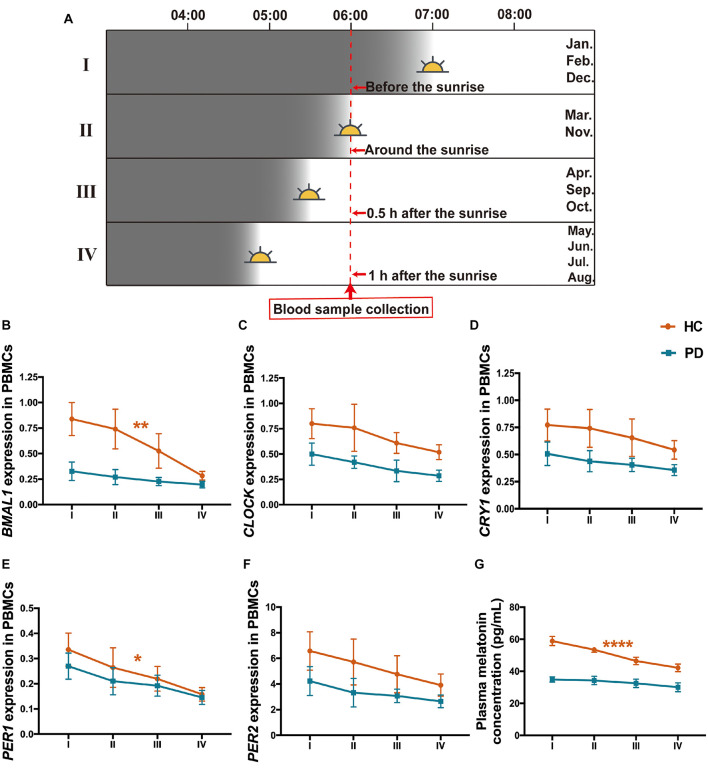
Expression levels of the five clock genes and melatonin in different sunlight intensity of HC and patients with PD. **(A)** A schematic diagram of the sunlight conditions when sampling in different groups of months. The time of blood sampling from the subjects was fixed at 6–7 a.m. Subjects were divided into 4 groups according to the local sunrise schedule. I: Sampling before the sunrise (January, February, and December, n (HC: PD) = 79: 80); II: Sampling around the sunrise (March and November, n (HC: PD) = 58: 64); III: Sampling 0.5 h after the sunrise (April, September, and October, n (HC: PD) = 57: 63); IV: Sampling 1 h after the sunrise (May, June, July, and August; n (HC: PD) = 120: 119). **(B–F)** PBMCs levels of *BMAL1* **(B)**, *CLOCK* **(C)**, *CRY1* **(D)**, *PER1* **(E)**, and *PER2* **(F)** expression in the group of PD and HC, with different intensities of sunlight. **(G)** Plasma melatonin concentration in PD and HC with different intensities of sunlight. The results are the mean ± SEM values. *P < 0.05, **P < 0.01, and ****P < 0.0001.

Since we collected the peripheral blood samples from the subjects in this study at a specific time which is 6--7 a.m. but in different months from January 1, 2018, to March 31, 2020, which means that there are variations of the environmental sunlight intensity when sampling. To clarify the alterations of the clock genes as the sunlight changes in different months, subjects were further divided into 4 subgroups according to the annual sunrise schedule of Dalian city, China,^1^ including I: Sampling before the sunrise (blood collection in January, February, and December), II: Sampling around the sunrise (blood collection in March and November), III: Sampling 0.5 h after the sunrise (blood collection in April, September, and October), IV: Sampling 1 h after the sunrise (blood collection in May, June, July, and August) (Figure 5A). The results showed that all these four subgroups in PD presented lower levels of *BMAL1*, *CLOCK, CRY1*, *PER1*, *PER2*, and melatonin than those in HC (Figures 5B–G). In the group of HC, PBMCs levels of *BMAL1* (*P* < 0.01), *PER1* (*P* < 0.05) and plasma melatonin (*P* < 0.0001) exhibited significant variations with the enhancement of the sunlight intensity. However, there was a lack of light-dependent variation in *BMAL1* (*P* = 0.41, Figure 5B), *PER1* expression (*P* = 0.19, Figure 5E), and melatonin concentration (*P* = 0.48, Figure 5F) in PD patients. Down-regulation of *CLOCK*, *CRY1*, and *PER2* expression with higher light intensity was also found in HC and PD patients; however, no statistically significance was found (Figures 5C,D,F).

## Discussion

In this study, we measured the expression profiles of five principal clock genes (*BMAL1*, *CLOCK*, *CRY1*, *PER1*, and *PER2*) in the PBMCs, as well as the melatonin concentration in the plasma of PD patients and HC simultaneously. To our knowledge, it was the first study to analyze the expression levels of five clock genes from a relatively large population of PD patients and controls. The main finding from this study is that the peripheral molecular clock is altered in PD, which could be potential markers for the sleep and wakefulness disturbances of PD. Furthermore, this study reported for the first time the loss of light-dependent variations of *BMAL1* and *PER1* levels in PD patients.

The circadian rhythm in mammals is cell-autonomous depending on transcriptional autoregulatory feedback loops organized by a core set of clock genes (Zhang S. et al., 2020). *BMAL1* is a central component of the mammalian circadian clock system (Yang et al., 2018). *CLOCK* as the first discovered mammalian clock gene, along with *BMAL1* can form a heterodimer (*BMAL1*: *CLOCK*) that binds to regulatory elements (E-boxes) sites and induces the expression of other clock genes, including *PER* and *CRY* (Trott et al., 2018; Cox and Takahashi, 2019). The heterodimerized *PER* and *CRY* in the cytoplasm translocate to the nucleus, where they inhibit *BMAL1*: *CLOCK*-mediated transcription (Cox and Takahashi, 2019). Overall, these interlocked transcriptional feedback loops participate in the molecular foundation of circadian rhythms.

Prior studies of clock genes in PD patients are scarce. Similar to our findings, Cai et al. (2010) reported lower PBMCs levels of *BMAL1* in PD (*n* = 68) compared with HC (*n* = 54). While Breen et al. (2014). found no significant difference between the *BMAL1* levels in PD and HC at 6 a.m., there was an increased expression of *PER2* at 4 a.m. in PD. This study also found that plasma melatonin levels were decreased in PD patients compared with controls, which is consistent with the previous reports (Bordet et al., 2003; Breen et al., 2014; Videnovic et al., 2014). Besides, expression levels of *CLOCK* and *CRY1* in PD patients showed a significant association with age in PD patients but not in HC. It has been reported that aging is associated with the disturbance of the circadian system resulting in an internal desynchronization of rhythm (De et al., 2018). We suspect that circadian abnormalities could be more sensitive to aging in PD patients than that in HC. Although previous studies reported that signaling mediated by the dopamine activity could promote the transcriptional capacity of the *BMAL1-CLOCK* complex (Landgraf and Oster, 2012), we found no significant difference among the four different pharmacotherapy groups of PD patients in this study. Our results suggest that the molecular basis of altered circadian rhythm gene expression in PD is more likely to be the consequence of PD pathology. One recent brain bank case-control study found the disease-related neuropathological changes in the SCN of PD patients, indicating the pathophysiological mechanisms underlying the PD pathology and circadian rhythm abnormalities (Pablo-Fenandez et al., 2018). On the other hand, it has been proposed that circadian disruption could also influence the neurodegenerative process in PD (Musiek and Holtzman, 2016). Most notably, the alterations of clock genes and melatonin levels can be observed in the patients who were in the early stage of PD or recent-onset without any PD treatment, and these alterations were not significantly related to the disease severity and duration of PD. Our result suggests that the dysregulation of the peripheral molecular clock could be an early sign for PD, which helps to explain why the circadian-related symptoms, for instance, RBD and autonomic dysfunction, occur in the prodromal phase of PD (Liu and Le, 2020).

One of the most prominent outputs of the circadian system is the synchronization of the sleep-wake cycle (Landgraf and Oster, 2012). Our data further demonstrate that the disturbances of sleep and wakefulness, which are mainly manifested as RBD symptoms, EDS, and poor night sleep quality, are common in PD patients. Numerous studies have revealed RBD to be an original symptom and anticipant biomarker of PD (Li and Le, 2019; Zhang F. et al., 2020). Until now, the association between RBD and PD in the regulation of the circadian rhythm is still not fully understood. Given the cost and facilities required for the gold standard-polysomnography (PSG) for the diagnosis of RBD, questionnaires that adequately screen for pRBD are useful for clinical studies (Dauvilliers et al., 2008). This study using RBDQ-HK evaluation of pRBD documents suggested that there may be interactions between circadian rhythm disruption and the pathology of PD even without RBD. However, PD patients with comorbid RBD may have a severer disturbance of molecular circadian rhythm.

This study also provides important evidence on the potential of peripheral clock genes and melatonin for the identification of sleep and wakefulness disturbances in PD patients. This is probably because the altered clock genes and melatonin level may cause a disruption to the neural circuitry controlling circadian rhythms, which in turn leads to disturbances in the sleep-wake cycle of PD patients. There’s plenty of evidence showing that clock genes and the sleep-wake cycle are tightly linked (Wulff et al., 2009). Study on the BMAL1-deficient mice illustrated reduced sleep-wake rhythmicity and increased sleep fragmentation (Laposky et al., 2005). Besides, the mice lacking CRY1 also illustrated sleep structure alteration, including an increase in non-REM time and EEG delta power (Wisor et al., 2002). Gene association studies in two independent populations reported an association between sequence variants of *CLOCK* and sleep duration (Allebrandt et al., 2010). Polymorphisms of the human *PER1* and *PER2* gene has also been found in association with abnormal sleep pattern (Toh et al., 2001; Carpen et al., 2006). In addition to the clock genes, Breen et al. also found that alterations in melatonin output were significantly associated with reduced slow-wave and REM sleep (Maggio et al., 2019). Our results indicate that combined *BMAL1*, *CLOCK*, *CRY1*, *PER1*, *PER2*, and melatonin could be potential biomarkers for evaluating the sleep-wake rhythm disturbances of PD patients, which may help improve the accuracy of the clinical assessment and subjective sleep questionnaires. Future studies are needed to further explore our observations using objective measurements of RBD, EDS, and sleep quality.

Although circadian rhythm is endogenous, it is also adapted to the local environmental factors called “zeitgeber,” and light is generally regarded as the most important zeitgeber (Aumann et al., 2016). This study demonstrated a loss of rhythmic expressions of *BMAL1*, *PER1*, and melatonin levels in PD with the intensity of environmental light changes. To our knowledge, only one study has analyzed the effect of photoperiod on the tyrosine hydroxylase and dopamine transporter immunoreactivities in the midbrain of people who died in summer vs. winter (Aumann et al., 2016). The density of tyrosine hydroxylase and dopamine transporter immunostaining positive neurons was higher in summer compared with winter, indicating the relationships between environmental photoperiod and the activity of midbrain dopaminergic neurons (Aumann et al., 2016). The reason for the clock gene alterations caused by changing seasonal patterns is still not clear and warrants further investigations. Our results indicate that the greater intensity of the light when sampling, the closer levels of clock genes and melatonin are seen between PD patients and HC. From this point of view, light exposure may have therapeutic effects on PD via regulating melatonin output and clock gene expressions and improving the consolidation of sleep-wake cycles.

## Conclusion

In a relatively large clinical sample, this study found that the expression levels of *BMAL1*, *CLOCK, CRY1*, *PER1*, and *PER2* in the PBMCs, as well as plasma melatonin levels of PD patients, are significantly decreased compared with HC. Sleep and wakefulness disturbances are common in these PD patients and are associated with all five clock genes and melatonin levels. PD patients with comorbid pRBD had a more severe disturbance of molecular circadian rhythm. We also discovered that a combination of *BMAL1*, *CLOCK, CRY1*, *PER1*, *PER2*, and melatonin could be a potential biomarker for PD comorbid RBD and EDS. Further, our findings indicate the necessity of strengthening and stabilize circadian rhythmicity through therapeutic intervention on clock genes and melatonin to improve the life quality of PD patients.

## Data Availability Statement

The raw data supporting the conclusions of this article will be made available by the authors, without undue reservation.

## Ethics Statement

The studies involving human participants were reviewed and approved by the Ethics Committee of The First Affiliated Hospital of Dalian Medical University (approval number: LCKY2014-29). The patients/participants provided their written informed consent to participate in this study.

## Author Contributions

TL and WL designed the project of this manuscript. TL, CC, YLi, NW, YY, and MA-N carried out all the experiments. TL, CC, CJ, and HY contributed to statistical analyses and results interpretation. TL, CJ, and WL contributed to the drafting of the manuscript. TL, YLe, JQ, and WL revised the manuscript. WL contributed to the research concept, research administration. All authors edited and approved the final version of the manuscript.

## Conflict of Interest

The authors declare that the research was conducted in the absence of any commercial or financial relationships that could be construed as a potential conflict of interest.

## Publisher’s Note

All claims expressed in this article are solely those of the authors and do not necessarily represent those of their affiliated organizations, or those of the publisher, the editors and the reviewers. Any product that may be evaluated in this article, or claim that may be made by its manufacturer, is not guaranteed or endorsed by the publisher.

## Footnotes

1
https://www.timeanddate.com/sun/china/luda
