# Supplementary material for: Peripheral Clock System Abnormalities in Patients With Parkinson’s Disease
Source: Front Aging Neurosci. 2021 Oct 1;13:736026. doi: 10.3389/fnagi.2021.736026 (PMC8519399; doi:10.3389/fnagi.2021.736026)
Supplement: Supplementary file 1 [file Data_Sheet_1.docx]

**Supplementary materials**

**Figure S1. The Influence of medications on the expression levels of *BMAL1*, *CLOCK*, *CRY1*, *PER1, PER2* and melatonin of PD patients.**

**
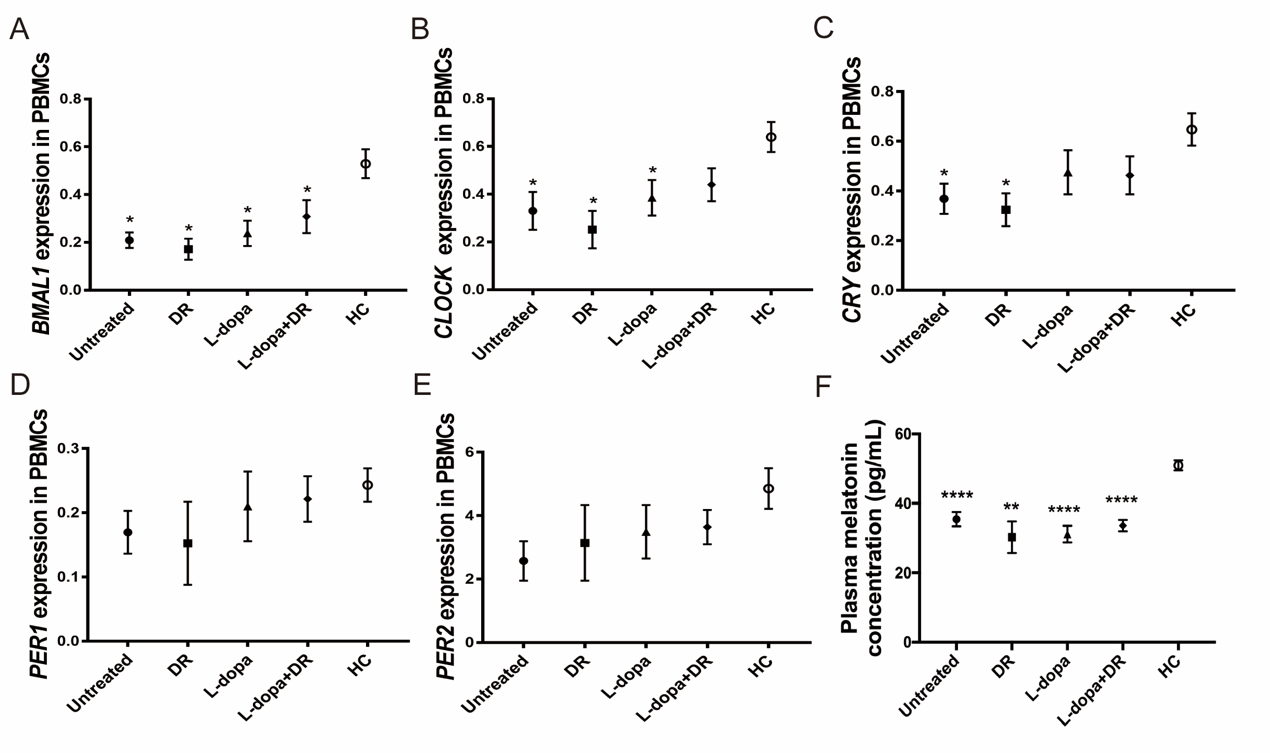
**

**(A-E):** The levels of *BMAL1* (**A**), *CLOCK* (**B**), *CRY1* (**C**), *PER1* (**D**), *PER2* (**E**) expression, and melatonin levels (**F**) in PD patients with different anti-PD medications and HC (n=314). The results are the mean ± SEM values. **P*< 0.05, relative to HC. Untreated, n=107; DR: DA receptor agonists monotherapy, n=66; L-dopa: l-dopa monotherapy, n=34; L-dopa+DR: the combination of DA agonists and l-dopa, n=119. Kruskal-Wallis test.

**Table S1. List of primers used for quantitative real-time PCR assays.**

| mRNA | Primers |
| --- | --- |
| *BMAL1* | Forward: 5’-TGGGGTCTTTCAGCTCACAC- 3’ |
|  | Reverse: 5’-GAGCTTCTTGGGATGGGTCT- 3’ |
| *CLOCK* | Forward: 5’-GACACAGGCGGGGTAGTG- 3’ |
|  | Reverse: 5’-CAAGTAAAAATTCGTGTGGCTTGC- 3’ |
| *CRY1* | Forward: 5’-CCCGTCTGTTTGTGATTCGT- 3’ |
|  | Reverse: 5’-TACTCCAGCTTCAGTTGCCA- 3’ |
| *PER1* | Forward: 5’-AACGGGCATGAGTCTAGAGG- 3’ |
|  | Reverse: 5’-GTAGGCAATGGAACTGCTGG- 3’ |
| *PER2* | Forward: 5’-GAAGGTGAAGGTCGGAGTC- 3’ |
|  | Reverse: 5’-CACCGCAAACATATCGGCAT- 3’ |
| *GAPDH* | Forward: 5’-GCGGTCACGTTTTCCACTATG- 3’ |
|  | Reverse: 5’-GAAGATGGTGATGGGATTTC- 3’ |
